# Real-life clinical practice results with vinflunine in patients with relapsed platinum-treated metastatic urothelial carcinoma: an Italian multicenter study (MOVIE-GOIRC 01–2014)

**DOI:** 10.1186/s12885-017-3466-3

**Published:** 2017-07-19

**Authors:** Rodolfo Passalacqua, Silvia Lazzarelli, Maddalena Donini, Rodolfo Montironi, Rosa Tambaro, Ugo De Giorgi, Sandro Pignata, Raffaella Palumbo, Giovanni Luca Ceresoli, Gianluca Del Conte, Giuseppe Tonini, Franco Morelli, Franco Nolè, Stefano Panni, Ermanno Rondini, Annalisa Guida, Paolo Andrea Zucali, Laura Doni, Elisa Iezzi, Caterina Caminiti

**Affiliations:** 1 Division of Oncology, ASST- Istituti Ospitalieri Cremona, Cremona, Italy; 2 Section of Pathological Anatomy, Polytechnic University of the Marche Region, School of Medicine, United Hospitals, Ancona, Italy; 3 Department of Urogynaecological Oncology, Istituto Nazionale per lo Studio e la Cura dei Tumori “Fondazione G Pascale”, IRCCS, Naples, Italy; 4 Oncologia Genitourinaria, Istituto Scientifico Romagnolo per lo Studio e la Cura dei Tumori (IRST) IRCCS, Meldola, Italy; 5 Oncologia, Istituti Clinici Maugeri, IRCCS, Pavia, Italy; 6 Oncologia Medica, Istituto Clinico Humanitas Gavazzeni, Bergamo, Italy; 7 Oncologia, IRCSS Ospedale San Raffaele, Milan, Italy; 8 Dipartimento di Oncologia, Università Campus Bio-Medico di Roma, Rome, Italy; 9 Ospedale Casa Sollievo della Sofferenza, San Giovanni Rotondo, Foggia Italy; 10 Oncologia, Istituto Europeo di Oncologia, Milan, Italy; 11 Oncologia, Arcispedale Santa Maria Nuova, IRCCS, Reggio Emilia, Italy; 12 Department of Oncology and Hematology, University of Modena and Reggio Emilia, Modena, Italy; 13 Oncologia, Humanitas Clinical and Research Hospital, Rozzano, Milan Italy; 14 Oncologia, Azienda Ospedaliera Universitaria Careggi, Florence, Italy; 15 Ricerca e Innovazione, Azienda Ospedaliero-Universitaria di Parma, Parma, Italy

**Keywords:** Vinflunine, Transitional cell carcinoma of the urothelium, Platinum-based chemotherapy, Real-life setting, Italian, Effectiveness

## Abstract

**Background:**

Vinflunine is the only chemotherapeutic agent shown to improve survival in platinum-refractory patients with metastatic transitional cell carcinoma of the urothelium (TCCU) in a phase III clinical trial, which led to product registration for this indication in Europe. The aim of this study was to assess the efficacy of vinflunine and to evaluate the prognostic significance of risk factors in a large, unselected cohort of patients with metastatic TCCU treated according to routine clinical practice.

**Methods:**

This was a retrospective multicenter study. Italian cancer centers were selected if, according to the Registry of the Italian Medicines Agency (AIFA), at least four patients had been treated with vinflunine between February 2011 and June 2014, after first- or second-line platinum-based chemotherapy. The primary objective was to test whether the efficacy measured by overall survival (OS) in the registration study could be confirmed in routine clinical practice. Multivariate analysis was carried out using Cox proportional hazard model.

**Results:**

A total of 217 patients were treated in 28 Italian centers. Median age was 69 years (IQR 62–76) and 84% were male; Eastern Cooperative Oncology Group performance status (ECOG PS) was ≥ 1 in 53% of patients. The median number of cycles was 4 (IQR 2–6); 29%, 35%, and 36% received an initial dose of 320 mg/m^2^, 280 mg/m^2^ or a lower dose, respectively. Median progression-free survival (PFS) and OS for the entire population was 3.2 months (2.6–3.7) and 8.1 months (6.3–8.9). A complete response was observed in six patients, partial response in 21, stable disease in 60, progressive disease in 108, with a disease control rate of 40%. Multivariate analysis showed that ECOG PS, number of metastatic sites and liver involvement were unfavorable prognostic factors for OS. Toxicity was mild, and grade 3–4 adverse effects were mainly: neutropenia (9%), anemia (6%), asthenia/fatigue (7%) and constipation (5%).

**Conclusions:**

In routine clinical practice the results obtained with VFL seem to be better than the results of the registration trial and reinforce evidence supporting its use after failure of a platinum-based chemotherapy.

**Electronic supplementary material:**

The online version of this article (doi:10.1186/s12885-017-3466-3) contains supplementary material, which is available to authorized users.

## Background

Urothelial cancer is the sixth most common type of cancer in the European Union and is responsible for 40,000 cancer-related deaths every year [1]. It is estimated that approximately 27,000 new cases of urothelial bladder cancer are diagnosed every year in Italy [2]. Muscle-invasive disease is among the most aggressive epithelial cancers. Radical cystectomy after neoadjuvant cisplatin-based chemotherapy as well as bilateral extended pelvic lymphadenectomy is the standard of care. However, about 50% of patients will relapse following surgery; the 5-year survival rate is approximately 60%, and 25–35% in high-risk patients (stages T3–4 and/or N+) [3, 4].

At present, a platinum-based chemotherapy is the standard front-line treatment in the metastatic setting. The combinations gemcitabine/cisplatin or methotrexate, vinblastine, adriamycin, and cisplatin (M-VAC) are used in patients able to tolerate cisplatin, while a carboplatin-based regimen or a single agent are the choice for the about 50% of patients unfit for a cisplatin-containing regimen. Although response rates are initially high, with about 50% reported in phase III trials, the majority of responding patients develop progressive disease within 8 months [5–7].

In small single-arm phase II trials, multiple traditional agents and novel targets have been studied in the second-line setting after a platinum-based regimen showing different overall response rates and median survivals [8, 9]. Due to limited therapeutic benefit, none of these treatments have been investigated in phase III trials.

Thus far, vinflunine is the only chemotherapeutic agent to have been studied in a randomized phase III trial [10] for the treatment of advanced or metastatic transitional cell carcinoma of the urothelium (TCCU) after failure of platinum-based chemotherapy. Vinflunine is a microtubule-targeting agent that induces mitotic arrest with subsequent cell death [11]; at non-cytotoxic concentrations, vinflunine also exerts antiangiogenic and antivascular activity [12]. The randomized phase III trial [10] demonstrated that after failure of a platinum-containing therapy in patients with metastatic disease, chemotherapy with vinflunine prolonged median overall survival (OS) by 2.6 months as compared to best supportive care (6.9 vs 4.3 months) with a 22% reduction in the risk of death, a statistically significant improvement, which was maintained in the eligible population in long-term (>3.5 years) follow-up, and manageable side effects [13].

Due to the favorable phase III results, vinflunine has been the only chemotherapeutic agent registered in Europe since 2009 for the treatment of advanced or metastatic TCCU after failure of platinum-based chemotherapy. An analysis of the data from the pivotal phase III study with vinflunine [14] and a retrospective analysis of pooled prospective phase II trials [15] produced interesting additional data; the main adverse prognostic factors for OS in patients who have failed a platinum-based regimen were hemoglobin <10 g/dL, the presence of liver metastases, performance status (PS) >0 and the time from prior chemotherapy (TFPC) <3 months; patients harboring a combination of all risk factors had a worse OS compared with those who had none.

After vinflunine entered the market, its effectiveness and good tolerability were confirmed in clinical practice by several observational studies performed in different European countries [16–22]; these studies reported a disease control rate (DCR) ranging from 30% to 65% and a median OS from 8 to 12 months.

In Italy, vinflunine has been marketed since February 2011; however, no data are available on its use in real-world clinical practice. The aim of this study was to investigate whether the results from a large cohort of unselected Italian patients treated with vinflunine were consistent with those of the phase III registration study [10] in terms of clinical outcome and safety.

## Methods

### Study design

This was a retrospective, observational multicenter trial, aiming to describe the activity and efficacy of vinflunine after a platinum-containing regimen in patients with advanced TCCU. All patients were treated and monitored according to local clinical practice. No additional procedures or patient visits other than usual clinical practice were planned for the study.

Italian cancer centers were selected if, according to the Registry of the Italian Medicines Agency (AIFA), at least four patients had been treated with vinflunine between February 2011 and June 2014. Participating centers are listed in Additional file 1.

### Participants

Eligibility criteria included age ≥ 18 years and administration of ≥1 vinflunine dose for metastatic or inoperable TCCU progressing after failure of a previous platinum-based chemotherapy regimen.

The patients included in the study received vinflunine according to local practice and in the best interest of the individual patient.

### Endpoints

The primary objective was to describe activity and efficacy measured as OS in a cohort of unselected patients treated in routine clinical practice. OS was defined as the interval between the date at which vinflunine treatment was initiated and the date of death from any cause or last follow-up visit.

Secondary objectives included other efficacy parameters, such as progression-free survival (PFS) and DCR, toxicity, clinical factors (sites of metastases, number of organs involved, hematochemical parameters, previous radiotherapy/chemotherapy, comorbidities, further lines of chemotherapy) and schedule of treatment (number of cycles, treatment duration, vinflunine initial and final doses, reasons for early discontinuation of treatment, supportive care). PFS was defined as the interval between the date at which vinflunine treatment was initiated and the date of disease progression, death in the absence of progression, or last follow-up for patients alive and progression-free at the time of last contact. Progression was defined as objective tumor progression, DCR was defined as the sum of complete response (CR), partial response (PR), and stable disease (SD), assessed in accordance with Response Evaluation Criteria in Solid Tumors criteria (RECIST Version 1.1). Response was assessed by the investigators in each single institution and no central revision of the responses was done.

### Data sources and measurement

Data were obtained retrospectively from patients’ medical records and no additional procedures/patient visits were planned in the study with respect to clinical practice. Data on demographic characteristics, treatment received since diagnosis, Eastern Cooperative Oncology Group (ECOG) PS, creatinine clearance and hemoglobin levels, sites of metastatic disease and vinflunine starting doses were collected.

Data were entered into an electronic case report form (e-CRF) by specifically trained staff. Both quality assurance activities (automatic checks) and monitoring activities of the centers’ progress were ensured. Periodic monitoring of center enrollment activity and data entry was performed (every three months, by phone and email). Study updates were shared among centers via a periodic newsletter. All e-CRF data were kept anonymous with respect to sensitive patient information by means of univocal identification codes (generated automatically through a hash function).

### Study size

Although this is a descriptive, non-comparative study, we performed sample size estimation to ensure estimate precision. The outcomes from the phase III clinical trial that led to the registration of vinflunine by the European Medicine Agency (EMA) [10] were used to determine the number of patients required to detect a similar-sized effect. Sample size was determined by considering a median OS ≤5.7 months (i.e. the lower limit of the 95% confidence interval [CI] of the OS obtained in the registration study) as the null hypothesis and a median OS ≥6.9 months (i.e. the OS obtained in the registration study) as the alternative hypothesis; with a difference in OS of 1.2 months, a one-tailed test, α 0.05 and 1-β of 80%, it was ascertained that at least 197 patients should be enrolled.

### Statistical methods

Summary descriptive statistics were applied to baseline characteristics. The Kaplan-Meier product limit method was used to estimate distributions of OS and PFS for all patients, and stratified by prognostic groups defined at therapy initiation or by other covariates of interest. Multivariate analysis was carried out using Cox proportional hazard model. Statistical analyses were performed using SAS (version 8.2) and in STATA/SE (version 11.0).

## Results

### Patient characteristics and clinical history

A total of 217 patients with metastatic TCCU progressing after failure of a previous platinum-based chemotherapy from 28 centers were enrolled in this study. Patients were followed up for a median of 7.43 (0.23–41.2) months. Patient characteristics and clinical history at baseline are summarized in Table 1. Two-thirds of the patients were aged ≥65 years, 84% were male and 53% had an ECOG PS ≥1, of which 7% had an ECOG PS = 2; liver metastases were present in 22% of patients with 53% overall having visceral disease. Clinical history showed that 7% of enrolled patients had previously received pelvic radiation, 2% neo-adjuvant chemotherapy and 22% adjuvant chemotherapy treatment.

**Table 1:** Patient characteristics and clinical history at baseline

	Present study	Registration study
Number of patients (*N*)	217	253
Gender, *n* (%)		
Male	183 (84)	NR
Female	34 (16)	NR
Age, years		
Median (IQR)	69 (62–76)	NR
< 65, *n* (%)	73 (34)	135 (53)
≥ 65, *n* (%)	144 (66)	118 (47)
ECOG PS, *n* (%)		
0	101 (47)	72 (28)
1	100 (46)	181 (72)
≥ 2	16 (7)	0 (0)
Creatinine clearance, n (%)		
> 60	125 (58)	134 (54)
40–60	75 (35)	104 (42)
< 40	17 (8)	10 (4)
Number of metastatic sites, n (%)		
1 site	127 (58)	62 (25)
≥ 2 sites	90 (42)	191 (75)
Visceral involvement, *n (%)*	115 (53)	187 (74)
Metastatic sites, *n* (%)		
Lymph nodes only	103 (47)	NR
Liver	47 (22)	NR
Lungs	70 (32)	NR
Bone	79 (36)	NR
Brain	2 (1)	NR
Other tissue (not visceral)	32(15)	NR
Prior pelvic irradiation, *n* (%)	16 (7)	57 (23)
Prior therapy with platinum-based regimen, *n* (%)		
Cisplatin	122 (56)	164 (65)
Carboplatin	91 (42)	75 (30)
Other platinum combination	4 (2)	14 (5)

*ECOG PS* Eastern Cooperative Oncology Group Performance Status, *IQR* interquartile range, *NR* not reported

All patients received at least one platinum-based regimen for metastatic disease prior to vinflunine; 122 (56%) were treated with cisplatin (either with gemcitabine or the M-VAC regimen), 91 (42%) with carboplatin plus gemcitabine, and 4 patients were treated with other platinum combinations.

### Vinflunine administration

With regard to vinflunine therapy, 167/217 (77%) and 50/217 (23%) of enrolled patients were treated as second or third line for metastatic TCCU, respectively, and 76/217 (35%) of patients were progressing less than 3 months from previous chemotherapy before starting vinflunine. Patients initially received 320 mg/m^2^ (29%), 280 mg/m^2^ (35%), 250 mg/m^2^ (24%) or 200 mg/m^2^ (12%) vinflunine every 21 days. During the study, 15 patients (10%) initially treated with 280 mg/m^2^ had a dose escalation to 320 mg/m^2^ and 39 patients (18%) had a dose reduction, mainly at the third cycle. The median number of cycles was 4 (interquartile range [IQR] 2–6). The reasons for vinflunine discontinuation were: progressive disease (70%), planned cycles (14%), toxicity (10%) and death before response evaluation (5%). Some patients received growth factors as a curative (11%) or prophylactic (20%) measure. Overall, 31% of patients received granulocyte-colony stimulating factor (G-CSF).

### Efficacy criteria

**Fig. 1:**
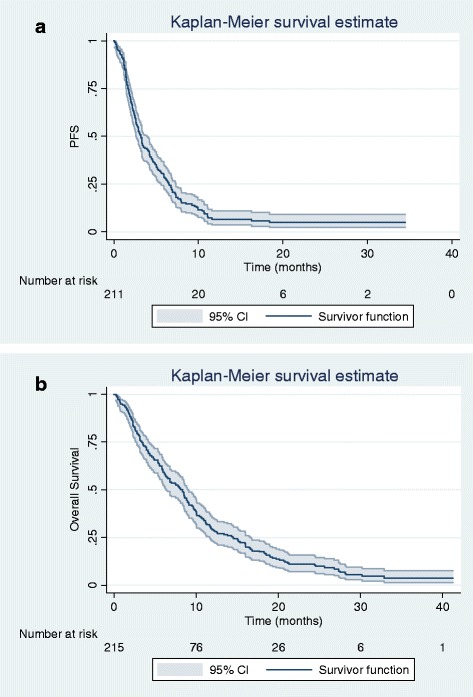
Kaplan-Meier curve for progression-free survival (PFS) (**a**), and overall survival (OS) (**b**) for patients with advanced or metastatic transitional cell carcinoma of the urothelium treated with vinflunine after failure of a platinum-based chemotherapy

Median PFS and OS for the entire population were 3.2 months (95% confidence interval [CI] 2.6–3.7) and 8.1 months (95% CI 6.3–8.9) (Fig. 1).

**Fig. 2:**
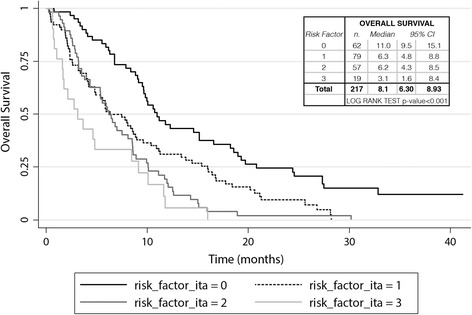
Kaplan-Meier curve for overall survival according to the number of risk factors including Eastern Cooperative Oncology Group performance status (ECOG PS), number of metastatic sites, and presence of liver metastasis

Both univariate and multivariate analysis showed that ECOG PS, number of metastatic sites and liver involvement were unfavorable prognostic factors for OS, whilst the same association was not observed for a hemoglobin level < 10 g/dL and TFPC <3 months (Table 2). At the time of the present analysis, 195 patients had died and 22 were alive; OS was negatively correlated with the number of risk factors (Fig. 2). CR was observed in 6 patients (3%), PR in 21 (10%), SD in 60 (28%) and PD in 108 (50%) patients, with a DCR of 40% (Table 3).

**Table 2:** Survival using univariate-multivariate analysis according to risk factors

					Multivariate		
	*n* (%)	Median (months)	[95% CI]	Log rank	HR	[95% CI]	*P*-value
Hemoglobin							
≥ 10	191 (88)	8.5	[6.8–9.6]	0.067			
< 10	26 (12)	4.8	[3.2–8.6]		1.45	0.93–2.25	0.097
ECOG PS							
0	101 (47)	9.8	[5.8–18.2]	<0.001			
≥ 1	116 (53)	5.6	[4.6–8.4]		1.68	1.26–2.25	<0.001
Number of metastatic sites							
1	127 (58)	9.5	[4.6–17.3]	<0.001			
≥ 2	90 (42)	5.8	[2.6–10.9]		1.48	1.08–2.03	0.014
Liver metastases							
No	170 (78)	8.6	[3.9–16.0]	0.004			
Yes	47 (22)	6.8	[3.1–8.5]		1.51	1.02–2.24	0.04
Visceral Involvement							
No	102 (47)	8.8	[3.9–15.0]	0.136			
Yes	115 (53)	7.5	[3.0–11.9]		1.03	0.75–1.42	0.839
Time from prior chemotherapy							
≥ 3 m	141 (65)	8.0	[5.9–8.9]	0.187			
< 3 m	76 (35)	8.1	[3.6–13.8]		1.19	0.88–1.62	0.264

*CI* confidence interval, *ECOG PS* Eastern Cooperative Oncology Group Performance Status, *HR* hazard ratio

**Table 3:** Efficacy of vinflunine treatment in patients with advanced or metastatic transitional cell carcinoma of the urothelium treated with vinflunine after failure of a platinum-based chemotherapy

Parameter	Present study	Registration study
*N* of randomly assigned patients	NA	253
*N* (%)	217 (100)	185 (73^a^)
Overall response, *n* (%)		
CR	6 (3)	0 (0)
PR	21 (10)	16 (9)
SD	60 (28)	86 (46)
PD	108 (50)	NR
DCR Not evaluable (or Missing data)	87 (40) 22 (10)	104 (41^b^) -
Objective response rate, *n* (%)	27 (13)	16 (9)
PFS, months		
Median [95% CI]	3.2 [2.6–3.7]	3.0 [2.1–4]
OS, months		
Median [95% CI]	8.1 [6.3–8.9]	6.9 [5.7–8.0]

*CR* complete response, *DCR* disease control rate, *OS* overall survival, *PD* progressive disease, *PFS* progression-free survival, *PR* partial response, *NR* not reported, *NA* not applicable

^a^Evaluable patients for response rate

^b^Calculated on Intention to treat population

Additional subset analyses of PFS/OS stratified by intensity of prior platinum regimen (e.g. cisplatin vs carboplatin), number of platinum courses received (<4 vs ≥4) showed no differences in OS or PFS in patients pretreated with carboplatin or cisplatin and according to the number of previous platinum cycles (see Additional file 1: Table S1).

### Safety

Adverse events are reported in Table 4. Briefly, the most commonly reported adverse events of any grade were fatigue/asthenia (24%), anemia (23%), constipation (22%) and neutropenia (15%). Grade ≥ 3 adverse events were neutropenia (9%), anemia (6%), asthenia/fatigue (7%), and constipation (5%). During vinflunine treatment, 63 (29%) patients received treatment for constipation as a curative measure and 106 (49%) as a prophylactic measure.

**Table 4:** Safety profile of vinflunine in patients with TCCU progressing following first or second line chemotherapy

Adverse events	Present study *N* = 217		Registration study *N*-253	
	All grades *n* (%)	Grade ≥ 3 *n* (%)	All grades *n* (%)	Grade ≥ 3 *n* (%)
Hematologic				
Neutropenia	33 (15)	19 (9)	190 (77)	123 (50)
Febrile neutropenia	7 (3)	7 (3)	15 (6)	15 (6)
Anemia	49 (23)	12 (6)	229 (93)	47 (19)
Thrombocytopenia	7 (3)	2 (1)	126 (51)	14 (6)
Non-hematologic				
Constipation	48 (22)	10 (5)	118 (48)	40 (16)
Stomatitis/mucositis	19 (9)	1 (0)	71 (29)	4 (2)
Fatigue/asthenia	52 (24)	16 (7)	124 (50)	48 (19)
Nausea	14 (6)	2 (1)	97 (39)	6 (2)
Vomiting	14 (6)	2 (1)	72 (29)	7 (3)
Neuropathy sensory	7 (3)	0 (0)	30 (12)	3 (1)
Abdominal pain	NA	NA	39 (16)	10 (4)
Alopecia	NA	NA	72 (29)	0 (0)
Death by toxicity		0 (0)		1 (0)

*NA* not available

## Discussion

With 217 patients enrolled from 28 Italian centers, the MOVIE study represents the largest-ever reported observational study evaluating vinflunine in nationwide clinical practice for the treatment of metastatic TCCU. The principal limitation of the present study is that, by design, the cohort was selected by receipt of vinflunine, and this may introduce a bias in comparison with a prospective randomized trial. However, in this study all patients consecutively followed in the participating centers were included over a well-defined period of time.

The characteristics of this population show that patients had mostly ECOG PS 0 and 1, but 7% had ECOG PS of 2. Vinflunine was used as third-line chemotherapy in 23% of patients as the study also included patients treated just after marketing authorization of vinflunine in Italy. Two thirds of the patients were ≥65 years old as opposed to less than half of the population enrolled in the registration study [10].

Overall, vinflunine resulted in an OS of 8.1 months, which is similar to OS reported in other published observational studies [16–22] (Table 5) and longer than the OS observed in the registration study (6.9 months) [10]. Compared to other published postmarketing observational studies, this study has some substantive differences: the sample size is larger and was calculated from the beginning on the basis of a solid statistical hypothesis, and we chose to include only patients treated during a well-defined time span and only in centers that had treated at least 4 patients, according to the AIFA register. These aspects reinforce the value of the results achieved.

**Table 5:** Observational retrospective real word multicenter studies

Author	No. Pts	PS (0–1)	Hb < 10 g/dl	Liver MTS	ORR	PFS (months)	OS (months)
Castellano [16]	102	92%	NR	17%	25%	3.9	10.0
Holmsten [18]	100	80%	39%	25%	23%	2.8	6.3
Medioni [19]	134	71%	24%	28%	22%	4.2	8.2
Pistamaltzian [20]	71	77%	22%	30%	13%	6.2	11.9
Retz [21]	77	100%	17%	22%	23%	NR	7.7
Present study	217	93%	12%	22%	13%	3.2	8.1

*No. Pts* number of patients, *PS* Performance status, *Hb* Haemoglobin, *MTS* metastasis, *ORR* overall response rate, *PFS* Progression Free survival, *OS* overall survival, *NR* Not reported

In line with the registration study, the present study confirms the role of ECOG PS ≥1; the number of disease sites and liver metastases as unfavorable prognostic factors for survival, whilst the same correlation was not observed for hemoglobin level < 10 g/dL and TFPC <3 months. We could postulate that the difference is probably related only to the sample size. In our population, only 12% of patients had Hb <10 g/dL whereas in the registration trial the basal value of Hb was not reported. Moreover, in our population, the median survival of this group was poor and was about half that of patients with basal Hb value > 10 g/dL (4.8 vs 8.5 months, respectively).

The 40% DCR is comparable with that of the registration study; however, a higher response rate was observed (13% vs. 9%), including CR in 3% of patients.

As reported in previous European observational studies [16–22], vinflunine had a manageable toxicity profile. In fact, the rates of grade 3–4 hematological and non-hematological adverse events were considerably lower than those reported in the registration study with about five times fewer cases of neutropenia and three times fewer cases of constipation. This difference could have been a result of some patients receiving prophylactic treatment for neutropenia and constipation. Indeed, treatment was better tolerated in the real-world setting, probably due to dose adaptation. Concerning drug exposure, the present real-world study shows that 64% of starting doses were 320 or 280 mg/m^2^, while 36% of initial treatments where started at 250 or 200 mg/m^2^. The median number of cycles was 4, higher than that reported in the phase III study.

The landscape for urothelial carcinoma treatment is rapidly changing with the introduction of immunotherapy and in particular checkpoint inhibitors targeting the programmed cell death protein (PD-1) pathway. Different agents targeting the PD-1 pathway have shown promising results in patients with metastatic urothelial cancer [23, 24]. The majority of these agents are currently under investigation in phase II or III clinical trials in the second- and first-line treatment of TCCU and recently the USA Food and Drug Administration (FDA) approved the first of such agents, atezolizumab, in patients with urothelial cancer progressing after a platinum-based chemotherapy. The efficacy of immunotherapy seems to be correlated with the ligand expression pattern on tumor cells and tumor-infiltrating immune cells assessed by immunohistochemistry, suggesting that treatments need to be tailored to patient subgroups with specific immunochemistry profiles [23]. Overall, median survival observed with immuno-checkpoint inhibitors is comparable to that observed with vinflunine in our study. The real impact of these treatments on OS and the best ways of integrating immunotherapy with existing chemotherapy treatments remain to be determined.

## Conclusions

The results of this study support those of other observational studies in confirming the efficacy of vinflunine in clinical practice, and its use in patients with metastatic urothelial cancer after failure of a platinum-based chemotherapy. Vinflunine is currently the only chemotherapeutic agent for which efficacy and clinical benefit have been confirmed in a real-life clinical practice setting in a large cohort of patients. Consequently, the Italian Association of Medical Oncology guidelines [2] have been updated and clinical recommendations amended in favor of vinflunine. Finally, a recently published European epidemiological survey (EPICURE Study) on the treatment attitude in patients having progressed on a platinum-based chemotherapy [25], showed that vinflunine was the preferred choice as second chemotherapy regimen after platinum-based chemotherapy, substantiating its status as standard therapy in Europe within the clinical practice setting.

## Additional file

Additional file 1: List of participating centers. **Table S1.** Stratified analysis of overall survival (OS) and progression-free survival (PFS) according to previous Cisplatin or Carboplatin treatment and to the number of cycles received. (DOCX 46 kb)

## Abbreviations

AIFA: Italian Medicines Agency
CI: Confidence interval
CR: Complete response
DCR: Disease control rate
ECOG: Eastern Cooperative Oncology Group
EMA: European Medicines Agency
FDA: Food and Drug Administration
IQR: Interquartile range
M-VAC: Methotrexate, vinblastine, adriamycin and cisplatin
OS: Overall survival
PFS: Progression-free survival
PR: Partial response
PS: Performance status
RECIST: Response Evaluation Criteria in Solid Tumors
SD: Stable disease
TCCU: Transitional cell carcinoma of the urothelium
TFPC: Time From Prior Chemotherapy
