# Supplementary material for: Real-life clinical practice results with vinflunine in patients with relapsed platinum-treated metastatic urothelial carcinoma: an Italian multicenter study (MOVIE-GOIRC 01–2014)
Source: BMC Cancer. 2017 Jul 19;17:493. doi: 10.1186/s12885-017-3466-3 (PMC5517798; doi:10.1186/s12885-017-3466-3)
Supplement: Additional file 1: — List of participating centers. Table S1. Stratified analysis of overall survival (OS) and progression-free survival (PFS) according to previous Cisplatin or Carboplatin treatment and to the number of cycles received. (DOCX 46 kb) [file 12885_2017_3466_MOESM1_ESM.docx]

**Additional file 1:**

ASST- Istituti Ospitalieri Cremona, Cremona, Italy

Ethical Committee: Comitato Etico Area Cremona Mantova e Lodi (Cremona)

Dipartimento di Oncologia, AUSL Piacenza, Italy

Ethical Committee: Comitato Etico della Ausl di Piacenza

Oncologia, Azienda Ospedaliera Universitaria Careggi, Firenze, Italy

Ethical Committee: Comitato Etico Area Vasta Centro

Oncologia, Humanitas Clinical and Research Hospital, Rozzano, Milan, Italy

Ethical Committee: Comitato Etico Indipendente IRCCS Istituto Clinico Humanitas Rozzano (Mi)

Oncologia, Istituto Europeo di Oncologia, Milan, Italy

Ethical Committee: Comitato Etico dell'IRCCS Istituto Europeo di Oncologia e Centro Cardiologico Monzino (Milano)

Oncologia Medica, Ospedale Regionale Bolzano, Italy

Ethical Committee: Comitato Etico del Comprensorio Sanitario di Bolzano

Oncologia Medica, Policlinico Universitario Campus Bio-Medico di Roma, Rome, Italy

Ethical Committee: Comitato Etico Campus Biomedico (CECBM) Roma

Oncologia, Arcispedale Santa Maria Nuova, IRCCS, Reggio Emilia, Italy

Ethical Committee: Comitato Etico Provinciale di Reggio Emilia

Oncologia, Policlinico di Modena, Modena, Italy

Ethical Committee: Comitato Etico Provinciale di Modena

Oncologia, AO Carlo Poma, Mantova, Italy

Ethical Committee: Comitato Etico Area Cremona Mantova e Lodi (Cremona)

Oncologia, Ospedale Santa Maria degli Angeli, Pordenone, Italy

Ethical Committee: Comitato Etico Regionale Unico (CERU)

Oncologia, Istituti Clinici Maugeri, IRCCS, Pavia, Italy

Ethical Committee: Comitato Etico della Fondazione Salvatore Maugeri Pavia

Oncologia, Istituto Clinico Humanitas Gavazzeni, Bergamo, Italy

Ethical Committee: Comitato Etico Indipendente IRCCS Istituto Clinico Humanitas Rozzano (Mi)

Oncologia, Ospedale ULSS, Bassano del Grappa, Vicenza, Italy

Ethical Committee: Comitato Etico per le Sperimentazioni Cliniche, Provincia di Vicenza

Oncologia, IRCSS Ospedale San Raffaele, Milan, Italy

Ethical Committee: Comitato Etico IRCCS San Raffaele Milano

Ospedale Casa Sollievo della Sofferenza, San Giovanni Rotondo, Foggia, Italy

Ethical Committee: Comitato Etico per la Fondazione Casa Sollievo della Sofferenza, San Giovanni Rotondo (Foggia)

Ricerca e Innovazione, Azienda Ospedaliero- Universitaria, Parma, Italy

Ethical Committee: Comitato Etico per Parma

UO Medicina Oncologica Ospedale Ramazzini, Carpi, Italy

Ethical Committee: Comitato Etico Provinciale di Modena

UO Oncologia Medica, Ospedale Santa Chiara AOU Pisana, Pisa, Italy

Ethical Committee: Comitato Etico Area Vasta Nord Ovest (CEAVNO)

UO Oncologia Medica Ospedale Santa Croce, Fano, Italy

Ethical Committee: Comitato Etico Azienda Ospedaliera Ospedali Riuniti Marche Nord

UO Oncologia Medica, Policlinico Sant'Orsola Malpighi, Bologna, Italy

Ethical Committee: Comitato Etico Indipendente dell'Azienda Ospedaliera Universitaria Policlinico S.Orsola Malpighi Bologna

UOC Oncologia Medica, Ospedale Misericordia, Azienda USL9, Grosseto, Italy

Ethical Committee: Comitato Etico Area Vasta Sud Est (CEAVSE)

UOC Oncologia Medica, Ospedale Versilia, Lido di Camaiore, Lucca, Italy

Ethical Committee: Comitato Etico Area Vasta Nord Ovest (CEAVNO)

UOC Oncologia Medica, Azienda Ospedaliera San Carlo, Milan, Italy

Ethical Committee: Comitato Etico Interaziendale Milano Area A

UOC Oncologia, ULSS 10 Veneto Orientale, San Donà di Piave, Venice, Italy

Ethical Committee: Comitato Etico per la Sperimentazione Clinica della Provincia di Venezia e IRCCS San Camillo (CESC)

UOSD Oncologia, Ospedale Civile di Avezzano, L'Aquila, Italy

Ethical Committee: Comitato Etico per le Province di L'Aquila e Teramo

Uro-Ginecologia, IRST Meldola-Presidio Ospedaliero Forlì, Meldola (FC), Italy

Ethical Committee: Comitato Etico di Area Vasta Romagna di Cesena e Istituto Scientifico Romagnolo per lo Studio e la Cura dei Tumori di Meldola (FC)

Uro-Ginecologia, Istituto Nazionale per lo Studio e la Cura dei Tumori “Fondazione G Pascale”, IRCCS, Naples, Italy

Ethical Committee: Comitato Etico IRCCS Istituto Pascale Napoli

**Additional file: Table S1.** Stratified analysis of overall survival (OS) and progression-free survival (PFS) according to previous Cisplatin or Carboplatin treatment and to the number of cycles received.

|  |  | **Carboplatin** | **Cisplatin** | **Nr of cycles <4** | **Nr of cycles ≥4** |
| --- | --- | --- | --- | --- | --- |
| **OS, months** | Median | 8.10 | 7.97 | 8.10 | 6.77 |
|  | 95% CI | 5.9 - 10.0 | 5.4 - 8.9 | 6.3 - 9.1 | 4.6 - 9.6 |
| **PFS, months** | Median | 3.27 | 2.60 | 3.27 | 2.57 |
|  | 95% CI | 2.8 - 4.6 | 2.1 - 3.9 | 2.8 - 4.2 | 2.1 - 3.7 |

|  |
| --- |
